# How ready are our health systems to implement prevention of mother to child transmission Option B+?

**DOI:** 10.4102/sajhivmed.v16i1.386

**Published:** 2015-10-07

**Authors:** Palesa Nkomo, Natasha Davies, Gayle Sherman, Sanjana Bhardwaj, Vundli Ramokolo, Nobubelo K. Ngandu, Nobuntu Noveve, Trisha Ramraj, Vuyolwethu Magasana, Yages Singh, Duduzile Nsibande, Ameena E. Goga

**Affiliations:** 1 Health Systems Research Unit, South African Medical Research Council, South Africa; 2 Wits Reproductive Health and HIV Institute, University of the Witwatersrand, South Africa; 3 National Institute for Communicable Diseases, Johannesburg, South Africa; 4 Department of Paediatrics and Child Health, University of the Witwatersrand, South Africa; 5 The United Nations Children's Fund, Pretoria, South Africa; 6 Department of Paediatrics, University of Pretoria, South Africa

## Abstract

In January 2015, the South African National Department of Health released new consolidated guidelines for the prevention of mother to child transmission (PMTCT) of HIV, in line with the World Health Organization's (WHO) PMTCT Option B+. Implementing these guidelines should make it possible to eliminate mother to child transmission (MTCT) of HIV and improve long-term maternal and infant outcomes. The present article summarises the key recommendations of the 2015 guidelines and highlights current gaps that hinder optimal implementation; these include late antenatal booking (as a result of poor staff attitudes towards ‘early bookers’ and foreigners, unsuitable clinic hours, lack of transport to facilities, quota systems being applied to antenatal clients and clinic staff shortages); poor compliance with rapid HIV testing protocols; weak referral systems with inadequate follow-up; inadequate numbers of laboratory staff to handle HIV-related monitoring procedures and return of results to the correct facility; and inadequate supply chain management, leading to interrupted supplies of antiretroviral drugs. Additionally, recommendations are proposed on how to address these gaps. There is a need to evaluate the implementation of the 2015 guidelines and proactively communicate with ground-level implementers to identify operational bottlenecks, test solutions to these bottlenecks, and develop realistic implementation plans.

## Introduction

### Context and summary of 2015 prevention of mother to child transmission guidelines

South Africa has the highest HIV incidence rates globally, and is the largest provider of antiretroviral therapy in the world.^1,2^ In January 2015, the South African National Department of Health (NDoH) released new national consolidated guidelines, including an approach akin to World Health Organization (WHO) Option B+ for the prevention of mother to child transmission (PMTCT) of HIV.^3^ These guidelines harmonise triple antiretroviral treatment (ART) regimens for infants and young children, adolescents, pregnant and breastfeeding women, and adults to facilitate continuity of care. The guidelines stipulate lifelong ART for all pregnant and breastfeeding women and HIV-positive infants regardless of their CD4 cell count.^3^ Box 1 summarises the main differences between the 2015^3^ and 2013^4^ PMTCT guidelines. Specific algorithms have been developed for women with comorbidities (e.g. active psychiatric illness, renal dysfunction and/or anaemia) and these remain unchanged compared with the 2013 South African guidelines.^3,4^ The 2015 guidelines highlight the need to improve access to testing and treatment in general, to achieve the 90/90/90 target (90% coverage for HIV testing, 90% coverage for ART uptake amongst HIV-positive patients, and viral suppression of 90% of patients on ART) and to prioritise HIV prevention and treatment amongst adolescents.^3^ Despite the complexity of the new policy, its ‘treatment as prevention’ approach amongst pregnant and breastfeeding women could move South Africa closer to achieving the fourth and fifth millennium development goals and the post-2015 sustainable development goals.

**BOX 1:** Key changes between the 2013 and January 2015 South Africa prevention of mother to child transmission guidelines.

2013 South African PMTCT guideline	New January 2015 South African PMTCT guideline
No mention of HIV testing amongst children.	Children aged ≥ 12 years may self-consent to an HIV test if they are of sufficient maturity to understand the benefits, risk and social implications.
**Re-testing of HIV-negative mothers or mothers of unknown HIV status:** should be tested for HIV at 6 weeks, 3 months, 9 months and 1 year postpartum, particularly if they are breast feeding.	**Re-testing of HIV-negative mothers:** 3-monthly through pregnancy at labour/delivery at 6-week infant immunisation visit (to identify newly exposed babies who need HIV testing) 12-weekly throughout breastfeeding till 24 months if breastfeeding continued.
**CD4 cell count ≤ 350 cells/μL used to guide eligibility for ART** amongst pregnant women without stage 3/4 disease or amongst non-pregnant HIV-positive patients with stage 3/4 disease.	**CD4 cell count not needed to determine ART eligibility amongst pregnant and lactating women:** Done for newly diagnosed patients at initiation to assess the need for: ART prioritisation (CD4 < 200 cells/μL) cotrimoxazole (CD4 < 200 cells/μL) tests to diagnose Cryptococcus infection (CD4 < 100 cells/μL). (Amongst the non-pregnant HIV-positive population, the threshold CD4 cell count for ART has been increased to ≤ 500 cells μL.)
CD4 cell count used for monitoring of ART at 12 months post initiation.	
**Initiate lifelong ART:** in all pregnant women with CD4 cell count ≤ 350 or stage 3/4 disease all HIV-positive children < 5 years old – immediately for infants and within 2 weeks for children between 1 and 5 years TB/HIV co-infected pregnant women. **Initiate ‘feeding-dependent’ ART** until 1 week after complete cessation of breastfeeding in women with CD4 > 350 without stage 3/4 disease.	**Initiate lifelong ART regardless of CD4 cell count for:** HIV-positive pregnant, breastfeeding women, or women within 1 year post partum for life HIV-positive women who attend for choice of termination of pregnancy (CTOP) (included in the 2015 PMTCT training package) HIV-positive children < 5 years (discussed in more detail in the paediatric guidelines) HIV/TB or HIV/hepatitis B co-infected women. **Duration of ART not dependent on feeding practice.**
Efavirenz (EFV) not used in first trimester of pregnancy amongst women on ART.	Efavirenz (EFV) used in first trimester of pregnancy amongst women on ART.
**Viral load monitoring** at first ANC if ART initiated before pregnancy and at 6 and 12 months post initiation.	**Viral load monitoring** at first ANC if ART initiated before pregnancy or within 3 months if ART initiated antenatally or during breastfeeding. Thereafter 6-monthly viral load monitoring.
**Daily infant nevirapine for 6 weeks** from as soon as possible post delivery.	As for 2013 plus **infant** **nevirapine could continue for 12 weeks if** maternal ART adherence has been suboptimal or maternal viral load > 1000 copies/mL or mother is newly diagnosed during breastfeeding. For newly diagnosed HIV-positive breastfeeding mothers, infant AZT and nevirapine should be initiated immediately. If infant PCR is negative, infant AZT can stop; but infant nevirapine should continue for 12 weeks. In HIV exposed infants < 18 months, infant nevirapine prophylaxis can be initiated if infant HIV rapid test result is not available or is positive, whilst awaiting infant's PCR result.
**Infant PCR testing to be conducted at:** birth in symptomatic infants failing to thrive (includes low birth weight, haematological abnormality such as anaemia or thrombocytopaenia, congenital pneumonia, hepatosplenomegaly, extensive oral candidiasis, significant lymphadenopathy, any opportunistic infections) 6 weeks in all HIV-exposed infants 6 weeks post cessation of breastfeeding if aged < 18 months and rapid HIV test if aged ≥ 18 months rapid HIV testing at 18 months. **Abandoned infants** should receive NVP < 72 hours post delivery and continued until HIV-exposure status has been determined. If the HIV rapid/ELISA test is positive, continue nevirapine until 6 weeks of age and do a PCR at 6 weeks. If the HIV rapid test result cannot be determined within 2 hours of encountering an abandoned baby, a stat dose of NVP is warranted.	**Infant PCR testing to be conducted at:** birth, or as soon as possible after birth amongst all HIV exposed infants 6 weeks in all HIV-exposed infants not testing positive at birth 10 weeks in infants not testing HIV positive at birth 16 weeks in infants receiving 12 weeks nevirapine 6 weeks post cessation of breastfeeding if aged < 18 months and rapid HIV tests if aged ≥ 18 months rapid HIV testing at 18 months for all HIV-exposed infants, for infants born to mothers of unknown HIV status, and for infants breastfed by a woman of unknown HIV status. For infants < 18 months, HIV rapid testing can be conducted to determine infant HIV-exposure. Abandoned infants – protocol as for 2013 guidelines.

*Source:* South African prevention of mother to child transmission Guidelines 2013 (South African antiretroviral treatment guidelines 2013. PMTCT guidelines: Revised March 2013 [cited 2015 Aug 27]. Available from http://www.sahivsoc.org/upload/documents/2013%20ART%20Guidelines-Short%20Combined%20FINAL%20draft%20guidelines%2014%20March%202013.pdf) and 2015 (South African National Department of Health. 2014 [cited 2015 Mar 02]. National consolidated guidelines for the prevention of mother-to-child transmission of HIV (PMTCT) and the management of HIV in children, adolescents and adults. Available from http://www.sahivsoc.org/upload/documents/HIV%20guidelines%20_Jan%202015.pdf)

PMTCT, prevention of mother to child transmission; ART, antiretroviral treatment; ANC, antenatal care; PCR, polymerase chain reaction; ELISA, enzyme-linked immunosorbent assay.

## Health systems’ readiness to implement the new guidelines

### System gaps that may hinder successful implementation of the 2015 South African prevention of mother to child transmission guidelines

In our opinion, there are five main requirements for successful implementation of the 2015 PMTCT guidelines: (1) early presentation at the health facility to access care (i.e. early antenatal booking), (2) universal antenatal HIV testing based on high-quality standardised operating procedures, with repeat testing of HIV-negative women, (3) immediate referral into appropriate care and retention in care, (4) adequate coverage of appropriate laboratory systems and (5) uninterrupted drug supplies. These require appropriate actions within the health system and amongst sufficiently informed and empowered individual mother-infant pairs. Box 2 presents a summary of the main health system and community gaps in optimal implementation of the 2015 PMTCT guidelines; these are explained in more detail below.

**BOX 2:** Summary of impediments to optimal prevention of mother to child transmission guideline implementation.

**1. Late antenatal booking:** poor staff attitudes towards ‘early bookers’ and foreigners unsuitable clinic hours lack of transport to facilities quota systems applied to antenatal clients clinic staff shortages and insufficient capacity.
**2. Poor quality HIV testing:** inadequate quality control poor supervision incomplete handling of discordant results poor data quality/documentation.
**3. Linkage and retention in care:** lack of service integration between (1) HIV-related care and routine maternal and child health services and (2) antenatal and postnatal services poor information systems and documentation hinder tracking those lost to follow-up weak referral systems.
**4. Laboratory capacity:** insufficient staff training limited staff capacity to handle increased demand as monitoring and numbers increase limited staff capacity to challenges with quickly communicating positive infant PCR results (e.g. facility Internet access and working telephones).
**5. Interrupted drug supplies:** inadequate supply chain management limited staff capacity to corruption.
**6. Community level:** late antenatal booking: > 50% book after 5 months’ gestation limited staff capacity to fear of HIV diagnosis limited staff capacity to stigma associated with HIV infection and with teenage pregnancy limited staff capacity to lack of demand for antiretroviral services owing to lack of awareness of benefits of treatment.

PCR, polymerase chain reaction.

### Late antenatal booking

Since 2001, South Africa has improved access to antenatal care, HIV testing and ART provision for pregnant women. Currently, antenatal care uptake is over 95%; HIV testing is offered by over 95% of health facilities, and more than 87% of HIV-positive pregnant women receive some form of ART.^3,5^ However, the District Health Information System shows that in 2011/2012 only 40.2% (range 33.6% in Eastern Cape to 56.2% in Western Cape) of pregnant women had their first antenatal booking visit before 20 weeks’ gestation, highlighting the first key bottleneck to successful guideline implementation.^6^ A study in North-West Province identified a variety of reasons for late booking, including late pregnancy disclosure amongst teenagers, fear of HIV testing, non-caring nurse attitudes, cultural beliefs that dissuade early revealing of pregnancy, lack of transport and unsuitable clinic opening hours.^6^ These findings were corroborated by research in Johannesburg which showed that 54% of pregnant women sought antenatal care later than 5 months’ gestation.^7^ Solarin and Black found that almost half of new mothers interviewed reported that their first antenatal booking was not accepted by health facilities for various reasons including (1) they needed ‘to make a booking appointment’, (2) they did not have a South African identity document and (3) clinics had reached their quota for the day.^7^ These obstacles delay first antenatal booking and thus HIV screening, ART initiation and detection of treatment failure amongst pregnant women on ART as recommended by the 2015 South African PMTCT guidelines.^3^

### Late HIV testing and poor quality HIV testing

There are grave concerns about quality control of HIV counselling and testing (HCT) at facilities, as shown by a study conducted in 455 sites (primary health care clinics, community healthcare centres and hospital gateway clinics) in Limpopo Province (Adrian Puren, personal communication, 11 March 2015). Poor quality control increases the risk of false-positive and -negative HIV results within the PMTCT programme. Concerns identified included inadequate training, frequent rotation of staff, lack of supervision and on-site quality control, incorrect storage of control samples, poor adherence to standard operating procedures (SOP) and improper stock control (Adrian Puren, personal communication, 11 March 2015). Anecdotal information gathered during healthcare provider (HCP) PMTCT guideline training found that HCPs are not waiting the required time before reading the HIV result, increasing the risk of false-negatives.

### Late referral into care and poor retention in care

Appropriate, timely referrals and linkages to care are needed antenatally and post delivery to facilitate uptake of and retention in care. Over 90% of facilities assessed during a national South African Medical Research Council (SAMRC) review conducted in 2010 had a referral system for infant and adult ART clients.^5^ Similarly, an Eastern Cape study found that 100% of facilities reported appropriate referral mechanisms for HIV-positive women.^8^ However, in the MRC review, 38% of facilities did not make appointments for their patients at referral centres, and only 50% of clinics followed up whether clients engaged with care at the referral site.^5^ From our research and clinical experience, postnatal loss to follow-up is particularly high in South Africa. Poor information systems and documentation contribute to difficulties with tracing such patients. In Malawi, loss to follow-up was five times higher for women who started ART during pregnancy and two times higher for women who started ART whilst breastfeeding than for women who started ART with WHO stage 3/4 disease or CD4 cell count ≤ 350 cells/µL.^9^ These data highlight the risk of poor retention in care postnatally and emphasise the need for robust referral systems, integrated services and effective tracking mechanisms to retain, or re-engage, women in ART care post delivery.

### Limited laboratory capacity

Laboratory capacity is needed for (1) viral load monitoring to identify poor adherence and treatment failure – a vitally important step in PMTCT programme success, (2) CD4 cell count to identify women who need cryptococcal antigen screening or cotrimoxazole prophylaxis, (3) routine antenatal bloods for ART toxicity monitoring and (4) repeated polymerase chain reaction (PCR) testing of HIV-exposed infants from birth (for symptomatic infants) to 18 months;^3^ laboratory capacity is accordingly a critical component of the PMTCT programme, and is key to timely referral into appropriate care. Previous challenges to implementing laboratory-based HIV tests in South Africa included difficulties processing large numbers of HIV-related specimens, high staff turn-over, and insufficient training in PCR techniques and CD4 measurements.^10^ Consequently, experienced staff carry the burden of training new staff^10^ and processing high quantities of specimens.

### Interrupted drug supplies

By mid-2014, an estimated 2.6 million people were on ART in South Africa.^3^ This number will further increase following the 2015 guideline implementation, creating additional demand for ART stock. Sustaining such ART programme expansion will necessitate more efficient, effective supply chain management and increased human resources. Yet the healthcare system remains plagued by frequent HIV medicines stock-outs and clinic staff shortages. Inadequate supply chain management and ‘corruption’ contribute to avoidable stock-out-related treatment interruptions,^11^ resulting in regimen modification at best or, at worst, drug discontinuation.^12^ Considering the complexity of the new guidelines in part reflects South Africa's mature HIV epidemic, including increasing rates of highly experienced ART patients with treatment failure and drug resistance. The effects of recurrent stock-outs on adherence, viral loads and drug resistance should not be underestimated.

## Recommendations

In light of the gaps identified above, we make several recommendations for optimal 2015 guideline implementation (Box 3).

**BOX 3:** Recommendations for optimal 2015 guideline implementation.

**Within the health system:** **Reduce the occurrence of late ANC booking:** provide standardised training regarding benefits of early booking for all women, regardless of nationality provide adolescent-friendly sexual and reproductive health services review clinic opening times and conduct local situational assessments to match the demand and supply of services review clinic accessibility (physically and opening hours) and public transport routes provision of more or more frequent mobile facilities/services review use of quota systems in antenatal clinics.
**Improve adherence to standard procedures for HIV testing:** improve the competence and expertise of all levels of staff conducting HCT establish supervision and monitoring systems for rapid HIV testing quality assurance.
**Strengthen linkages retention in care by establishing tracking systems:** strengthen service integration establish patient tracking systems, utilising ward-based outreach teams, unique patient identifiers and/or e-systems such as MomConnect improve referral systems with pre-booking and feedback, using a unique identifier and engaging District Specialist Teams.
**Increase laboratory capacity and reduce laboratory transport/feedback systems to reduce turnaround time:** increase staffing levels and training improve communication (working telephones, computers, Internet access) to expedite results access.
**Improve stock monitoring mechanisms and reduce drug stock-outs:** ensure that all facilities are trained in stock management, re-ordering procedures and lag times to avoid ART stock-outs increase capacity at facility level to use DHIS data to identify gaps and address them through quality improvement processes facility managers and district coordinators to be held accountable where the PMTCT programme and maternal and child health outcomes are suboptimal.
**Community level:** Improve awareness on importance of uptake of early antenatal care Eliminate prejudice and discrimination by healthcare workers against people who test HIV-positive, and educate communities about the importance of knowing one's HIV status Advocate at community levels to reduce fear and stigma around HIV and teenage/unwanted pregnancy and educate HIV-infected women regarding the treatment they should be able to access/demand Increase awareness about PMTCT/antenatal and postnatal care at community level

ANC, antenatal care; HCT, HIV counselling and testing; ART, antiretroviral treatment; DHIS, District Health Information System; PMTCT, prevention of mother to child transmission.

## Conclusion

The January 2015 PMTCT guideline recommendations are of a very high standard and based on the best intentions to improve the management of both HIV-positive and HIV-negative women. By implementing these guidelines, it should be possible to eliminate MTCT, improve maternal and infant outcomes, and ensure that women remain virologically suppressed and engaged in lifelong ART care. However, the implementation challenges might have been underestimated. Evaluation of the implementation process is needed to identify key bottlenecks and develop realistic implementation plans. A proactive process of communicating with ground-level implementers is needed to understand their challenges and to address these through well recognised, quality improvement processes.
